# Using the needs of WHO to prioritise Cochrane reviews: The case of antipsychotic drugs

**DOI:** 10.1186/1752-4458-5-25

**Published:** 2011-09-24

**Authors:** Marianna Purgato, Corrado Barbui, Clive E Adams

**Affiliations:** 1 Department of Public Health and Community Medicine, Section of Psychiatry and Clinical Psychology, University of Verona, Verona, Italy; 2 Division of Psychiatry, University of Nottingham, Nottingham, UK

## Abstract

**Background:**

This study aimed to investigate existing trialling activity relating to three antipsychotic drugs from the WHO List of Essential Medicines (chlorpromazine, fluphenazine decanoate, haloperidol), link existing trials to existing Cochrane reviews, identify gaps in reviewing activity on accessible treatments for people with schizophrenia.

**Methods:**

We used the Cochrane Schizophrenia Group's register searching for all studies comparing the three antipsychotic drugs with each other and with all other pharmacological interventions listed on the Essential Medicines List (with the addition of 'placebo or no drug'). For each we also considered studies that focussed on administration, dose, withdrawal and use of that drug in specific circumstances administration. Data were then extracted on a number of studies, number of participants within those studies, and as to whether a maintained review already exists. Finally, every effort was made to consider as to whether there were possibilities for missing comparisons that no one had ever investigated.

**Results:**

There has been considerable research activity involving the three 'essential' antipsychotics and also comparing those three drugs to others on the 'essential' list. We found 490 studies with 77957 participants for haloperidol, 316 studies with 29179 participants for chlorpromazine and 33 studies with 4503 participants for fluphenazine decanoate. Reviewing activity has also been considerable in this area but there are notable omissions which would necessitate new reviews to comprehensively cover the area.

**Conclusions:**

We have used the 'sample frame' of the WHO Essential drug list as a starting point. WHO prioritises for us those drugs that have universal accessibility but they may not be the compounds that are first choice if others are available. It is encouraging to see how many maintained reviews already exist to service those undertaking WHO guidelines. The needs of those guiding care can be taken as a means of prioritising research. For largest global impact WHO Essential Medicine list provides clear direction. By using this technique workload can be anticipated, prioritising can take place for new reviews and updates.

## Background

The Cochrane Collaboration is a unique organisation producing as well as maintaining systematic reviews, mostly focussed on the effects of healthcare interventions [1]. These reviews are regularly maintained in the light of new evidence or valid criticism. The Cochrane Library has been shown to contain the highest grade reviews of any publication [2].

With the enormous task of summarising the effects of all healthcare interventions (and maintaining those summaries) it is a logistical, ethical and moral dilemma where to focus efforts. Prioritising in the midst of so much of a 'confusion of evidence' has, for some time, troubled the Collaboration as a whole [3].

Mental health is no exception. The randomised controlled trial is the most powerful means of evaluating mental health treatments [4]. The five mental health Cochrane groups currently produce and maintain 512 reviews on the Cochrane Library but these remain a small fraction of the potential numbers of relevance and interest. With limited resources it is imperative that energies are not dissipated in producing reviews that are likely to be of little value to people with mental health problems. A series of competing priorities often results in continuation of the status quo - where the priorities of reviewer, or the editorial base, take precedence over others.

The recently produced WHO guidance on the care of people with mental health problems offered an opportunity to explore a technique for prioritisation [5]. 80% of the world's population with schizophrenia live in low and middle income countries. The mainstay of treatment of people with serious mental illnesses such as schizophrenia remains antipsychotic drugs but many of these drugs are unavailable worldwide or are available only to the rich.

WHO, have drawn up a list of drugs they designate as 'essential' [6]. "Essential medicines are those that satisfy the priority health care needs of the population. They are selected with due regard to public health relevance, evidence on efficacy and safety, and comparative cost-effectiveness. Essential medicines are intended to be available within the context of functioning health systems at all times in adequate amounts, in the appropriate dosage forms, with assured quality and adequate information, and at a price the individual and the community can afford."[6] This list includes over 350 medicines of which twelve are for mental and behavioural disorders: amitriptyline and fluoxetine for depressive disorder; carbamazepine, lithium carbonate and valproic acid for bipolar disorder, diazepam for anxiety disorder; clomipramine for obsessive-compulsive disorder; nicotine replacement therapy and methadone for disorders due to psychoactive substance use. The list includes only three drugs for psychotic disorder: chlorpromazine, haloperidol, and fluphenazine decanoate.

In these years several applications have been submitted to WHO to include other drugs in the Essential List of Medicine. The list of rejected drugs includes 51 pharmacological interventions of which 12 are psychotropic drugs (6 antipsychotics, 4 antidepressants, 1 anticonvulsant, 1 benzodiazepine) [7]. The reasons for rejection are not clear. Regarding antipsychotics, for example, Clozapine is mentioned in the WHO Intervention guide [5] for "treatment resistant schizophrenia" (those who have not responded to other antipsychotic agents at adequate dosages for adequate duration) but, nevertheless, appears in the rejected drugs list. Rejection could be from a series of factors such as evidence, cost, distribution, or/and ease of use.

In 2010, those drawing up the guidance for WHO contracted the Cochrane Schizophrenia Group to produce summaries of the findings of reviews relevant to these drugs, using the Grading of Recommendations, Assessment, Development and Evaluation (GRADE) approach [8]. This methodology is suitable for summarizing the evidence extracted from systematic reviews and meta-analyses into ''Summary of Findings (SoF) tables''; grading the quality of evidence summarized in SoF tables; and grading the strength of treatment recommendations [9].

This resulted in approximately forty summaries of findings from existing best evidence in Cochrane reviews. It also highlighted the possibility that some useful data may not have been systematically reviewed and therefore were not available to policy makers with global impact.

## Aims

To use WHO 'essential' antipsychotic drugs as a sampling frame, investigate existing trialling activity relating to each of these antipsychotic drugs, link existing trials to existing Cochrane reviews. We aim at identifying gaps in reviewing activity on accessible treatments for people with schizophrenia, in order to prioritise Cochrane reviews using the needs of WHO.

## Methods

We used the Cochrane Schizophrenia Group's register that contains 16,000 citations to approximately 13,000 studies. This register is complied of regular searching of 71 databases and is considerably more comprehensive that can be found either on Medline, PsycInfo, EMBase, or even the Cochrane Central Register of Trials [10]. This represents the most comprehensive register of subject-specific trials in existence. These studies are reliably indexed [11] regarding the intervention and the number of participants.

MP systematically searched the register for all studies comparing the three antipsychotic drugs with each other and with all other pharmacological interventions listed on the Essential Medicines List (with the addition of the intervention of 'placebo or no drug'). For each we also considered studies that focussed on administration, dose, withdrawal and use of that drug in specific circumstances administration.

Data were then extracted on a number of studies, number of participants within those studies, and as to whether a maintained review already exists. Finally, every effort was made to consider as to whether there were possibilities for missing comparisons that no one had ever investigated.

## Results

There has been considerable research activity involving the three 'essential' antipsychotics (Table 1) and also comparing those three drugs to others on the 'essential' list (Table 2). We found a total of 490 studies with 77957 participants for haloperidol, 316 studies with 29179 participants for chlorpromazine and 33 studies with 4503 participants for fluphenazine decanoate. Reviewing activity has also been considerable in this area but there are notable omissions which would necessitate new reviews to comprehensively cover the area.

**Table 1:** WHO three essential antipsychotics and possible comparisons

	RCTs	Total number of people	Cochrane review	Reference	Up to date?
**Chlorpromazine**					

*vs *fluphenazine depot	7	1314	✓	[13]	✗
*vs *haloperidol	51	6001	✓	[14]	✗
*vs *placebo	233	19854	✓	[15]	✗
Withdrawal	1	32	✓	[16]	✗
Techniques of administration	8	669	✗		
Dose	15	1279	✓	[17]	✗
In specific circumstances	1	30	✓	[18]	✗
**Fluphenazine Decanoate**					

*vs *chlorpromazine	7	1314	✓	[13]	✗
*vs *haloperidol	19	2339	✓	[13]	✗
*vs *placebo	4	456	✓	[13]	✗
Withdrawal	0	0	✗		
Techniques of administration	0	0	✗		
Dose	3	394	✗		
In specific circumstances	0	0	✗		
**Haloperidol**					

*vs *chlorpromazine	51	6001	✓	[14]	✗
*vs *fluphenazine depot	19	2339	✓	[13]	✗
*vs *placebo	237	41321	✓	[19]	✗
Withdrawal	3	126	✗		
Techniques of administration	50	10583	✗		
Dose	126	16470	✓	[20]	✗
In specific circumstances	4	1117	✓	[21]	✗

RCTs: Randomized Controlled Trials

Vs: Versus

✓: Systematic Reviews or updates already exist

✗: Systematic Reviews or updates do not exist

**Table 2:** WHO three essential antipsychotics and other drugs on the 'Essential' list (by drug class) to which they have been compared within randomized trials

	RCTs	Total number of people	Cochrane review	Reference	Up to date?
**Chlorpromazine**					
*vs *anticonvulsant agents	2	118	✗		
*vs *antidepressants	12	732	✓	[22,23]	✗
*vs *antihypertensive agents	8	286	✗		
*vs *antiparkinsonian agents	3	80	✗		
*vs *antituberculosis agents	1	25	✗		
*vs *barbiturates	3	750	✗		
*vs *benzodiazepines	9	457	✓	[24,25]	✗
*vs *mood stabilizers	2	49	✓	[26-28]	✗
*vs *opioid agonist	1	340	✗		
*vs *opioid antagonist	1	60	✗		
**Fluphenazine decanoate**					

*vs *antihypertensive agents	1	17	✗		
*vs *antituberculosis agents	1	55	✗		
**Haloperidol**					

*vs *antiarrhythmic agents	2	30	✗		
*vs *antidepressants	10	774	✓	[22,23]	✗
*vs *antidopaminergic agents	4	146	✗		
*vs *antigout agents	1	46	✗		
*vs *antihypertensive agents	3	122	✗		
*vs *antineoplastic agents	3	101	✗		
*vs *antiparkinsonian agents	8	265	✗		
*vs *antiprogesterone agents	1	80	✓	[29]	✗
*vs *benzodiazepines	35	4012	✓	[24,25]	✗
*vs *ethanol	1	35	✗		
*vs *glucose	1	unclear	✗		
*vs *mood stabilizers	23	1648	✓	[26-28]	✗
*vs *stimulant agents	1	20	✗		

RCTs: Randomized Controlled Trials

Vs: Versus

✓: Systematic Reviews or update already exist

✗: Systematic Reviews or update do not exist

## Discussion

We have used the 'sample frame' of the WHO Essential drug list as a starting point. WHO prioritises for us those drugs that have universal accessibility but they may not be the compounds that are first choice if others are available. Also, we have not considered comparisons of drugs with other interventions such as psychotherapies, techniques of management, and other physical treatment such as electro-convulsant therapy. Studies relevant to these comparisons do exist and recognise that the scope of our list of comparisons is limited. However, these other treatments are not listed by WHO as essential (except for treatment of every person with respect and dignity), they are not known to be universally accessible, and they are more difficult to define for global purposes of comparison. Confining the list to drugs only makes this list of comparisons serviceable. There may well be, therefore, other comparisons with universally accessible [non-pharmacological] treatments that we have not considered.

The key antipsychotics have been compared with many other drugs that fall into the broad categories seen within Table 2. We have not attempted to categorise every possible combination as we did for Table 1. Clearly researchers have undertaken many trials of treatments, even if used in a less than conventional way, using the three essential antipsychotics and compared them with other drugs. Many of these comparisons would seem odd or unusual but in situations where very few alternatives exist some less conventional alternative treatments may be considered. These have been much less commonly considered by reviewers and this exercise has highlighted that some should be the focus of reviews of the future.

It is encouraging to see how many maintained reviews already exist to service those undertaking WHO guidelines. This effort has been because of the foresight of reviewers, editors and sometimes funders (for the larger of the reviews). Most reviews, however, are not fully up-to-date. Even with reviews of older drugs and, therefore, less active areas of research, there remains the challenge of update. We expect that new fresh reviews meeting the needs of WHO and updates of not fully up-to-date reviews will be used to regularly update the WHO list of essential medicines and other lists of medicines included in formularies or emergency kits [12].

New techniques are being introduced to the science of data synthesis that allow use of more of the older data or at the very least their reconsideration. These include more explicit and transparent evaluation of potential risk of bias in the design of included studies, the possibility to explore the diversity of the results of different studies included in the analysis (heterogeneity), and the possibility to summarize the results of systematic reviews and meta-analyses into ''Summary of Findings (SoF) tables''.

## Conclusions

The needs of those guiding care can be taken as a means of prioritising research. For largest global impact WHO Essential Medicine list provides clear direction. By using this technique workload can be anticipated, prioritising can take place for new reviews and updates.

## Competing interests

The authors declare that they have no competing interests.

## Authors' contributions

MP, CEA and CB designed the study. MP extracted data, CEA analysed and interpreted data. MP and CEA drafted the first manuscript. CB commented and refined the manuscript in preparation for submission. All authors approved the final version to be published.
